# Occupational burnout and job satisfaction among physicians in times of COVID-19 crisis: a convergent parallel mixed-method study

**DOI:** 10.1186/s12889-021-10897-4

**Published:** 2021-04-28

**Authors:** Hamzeh Mohammad Alrawashdeh, Ala’a B. Al-Tammemi, Mohammad Kh. Alzawahreh, Ashraf Al-Tamimi, Mohamed Elkholy, Fawaz Al Sarireh, Mohammad Abusamak, Nafisa M. K. Elehamer, Ahmad Malkawi, Wedad Al-Dolat, Luai Abu-Ismail, Ali Al-Far, Imene Ghoul

**Affiliations:** 1 Sharif Eye Centers, Irbid, Jordan; 2 Department of Family and Occupational Medicine, Faculty of Medicine, University of Debrecen, Debrecen, Hungary; 3 Doctoral School of Health Sciences, University of Debrecen, Debrecen, Hungary; 4 Department of Special Surgery, Division of Urology, Al Bashir Hospital, Ministry of Health, Amman, Jordan; 5 Department of Radiology, King Hussein Hospital, Jordanian Royal Medical Services, Amman, Jordan; 6 Department of Epidemiology and Global Health, Faculty of Medicine, Umeå University, Umeå, Sweden; 7 Department of Ophthalmology, College of Medicine, Mutah University, Karak, Jordan; 8 Department of General and Special Surgery, Division of Ophthalmology, Faculty of Medicine, Al Balqa Applied University, Salt, Jordan; 9 Department of Public Health and Epidemiology, Faculty of Medicine, University of Debrecen, Debrecen, Hungary; 10 Faculty of Public and Environmental Health, University of Khartoum, Khartoum, Sudan; 11 School of Nutrition and Translational Research in Metabolism (NUTRIM), Department of Health Promotion, Maastricht University Medical Center, Maastricht University, Maastricht, The Netherlands; 12 Department of Ophthalmology, Faculty of Medicine, Yarmouk University, Irbid, Jordan; 13 Department of Clinical Medical Sciences, Faculty of Medicine, Yarmouk University, Irbid, Jordan; 14 Department of Orthopedic and Trauma Surgery, Jordan Hospital, Amman, Jordan; 15 Department of Pediatrics, Ibn Al Haytham Hospital, Amman, Jordan

**Keywords:** COVID-19 pandemic, Physicians, Occupational burnout, Job satisfaction, Jordan, Psychological distress, Physical burden, Mixed-method, Convergent parallel

## Abstract

**Background:**

Healthcare professionals including physicians were subjected to an increased workload during the COVID-19 crisis, leaving them exposed to significant physical and psychological distress. Therefore, our present study aimed to (i) assess the prevalence of burnout and levels of job satisfaction among physicians in Jordan, and (ii) explore physicians’ opinions, experiences, and perceptions during the pandemic crisis.

**Methods:**

This was a mixed-method study that utilized a structured web-based questionnaire and semi-structured individual interviews. The 10-Item Burnout Measure-Short version (BMS), and the 5-Item Short Index of Job Satisfaction (SIJS) were adopted to assess occupational burnout and job satisfaction, respectively. Semi-structured interviews were conducted, based on a conceptual framework that was developed from Herzberg’s Two-Factor Theory of Motivation and Job Demands-Resources Model. Descriptive statistics and regression models, as well as inductive thematic analysis, were used to analyze quantitative and qualitative data, respectively.

**Results:**

A total of 973 survey responses and 11 interviews were included in our analysis. The prevalence of burnout among physicians was (57.7%). Several significant factors were positively associated with burnout, including female gender, working at highly loaded hospitals, working for long hours, doing night shifts, lack of sufficient access to personal protective equipment, and being positively tested for SARS-CoV-2. Regarding job satisfaction, regression analysis revealed that age was positively associated with higher levels of job satisfaction. On contrary, being a general practitioner or specialist, working at highly loaded hospitals, low salaries, and suffering from burnout have predicted lower levels of job satisfaction.

Besides, four themes have emerged from the thematic analysis: (i) Work-induced psychological distress during the pandemic, (ii) Decision-driven satisfactory and dissatisfactory experiences, (iii) Impact of the pandemic on doctor-patient communication and professional skills, and (iv) Economic impacts of the pandemic crisis and lockdown.

**Conclusion:**

A significant physical and psychological burden was associated with the COVID-19 pandemic. Reliable efforts should be implemented aiming at protecting physicians’ physical and mental wellbeing, enhancing their working conditions, and raising awareness about burnout. Evidence-based decisions and proper utilization of financial and human resources at institutional and national levels are believed to be crucial for the sustainability of the health workforce, especially in crises.

**Supplementary Information:**

The online version contains supplementary material available at 10.1186/s12889-021-10897-4.

## Introduction

In December 2019, multiple cases of pneumonia of unknown origin that share similar presentation were reported in Wuhan city, Hubei province of China. The immense scientific efforts directed to find out the causative agent behind this clinical entity revealed a novel single-stranded RNA virus was behind these cases [1, 2]. The virus was named Severe Acute Respiratory Syndrome Coronavirus 2 (SARS-CoV-2). Later, this acute respiratory illness was referred to as Coronavirus Disease 2019 or COVID-19.

The extensive and rapid spread of the COVID-19 on a global scale has prompted the World Health Organization (WHO) to characterize it as a global pandemic in March 2020 [3]. As of April 19, 2021, this global pandemic has already resulted in more than 141 million confirmed cases and the death toll surpassed 3 million deaths globally [4]. Jordan, a middle-income country located in the Middle East, was also afflicted by the pandemic, and this has forced the government to impose various stringent control strategies to contain its spread [5]. A nationwide lockdown for approximately 2 months and a half was enforced by the Jordanian government as one of the control measures. The lockdown alongside other declared measures has placed the general population including healthcare workers under a new experience that possessed social, emotional, psychological, and financial impacts [6–9]. As of April 19, 2021, the total number of confirmed COVID-19 cases, active cases, and deaths in Jordan were 689,482, 36,688, and 8308, respectively, with a surge since early September 2020 [10, 11]. The sudden and accelerated rise in the number of COVID-19 cases has imposed an additional burden on decision-makers, healthcare professionals (HCPs) as well as the general population in the country [11].

The COVID-19 pandemic as a global public health issue has impacted most life domains and sectors, in which the health sector was not an exception. For example, In Italy and many other countries, the novel coronavirus SARS-CoV-2 has affected healthcare operators within the hospital and non-hospital settings [12, 13]. The figures, however, underestimate the current COVID-19 infection rate in healthcare settings, because healthcare workers with mild or asymptomatic infections might be less likely to be tested [14]. However, the COVID-19 infection can be recognized as an occupational injury and hazard among HCPs [15]. In an Italian study during the COVID-19 crisis, around 71.1% of physicians (Anesthetists) who participated in the study reported high levels of work-related stress with modest organizational justice and decreased levels of activities that enhance resilience and coping [16]. In the same study, the prevalence of insomnia, anxiety, and depression were around 36.7, 27.8, and 51.1%, respectively [16]. Apparently, HCPs including physicians and nurses were subjected to an increased workload as well as occupational distress during pandemic crises, leaving them vulnerable to suffer from burnout [17–19]. Burnout is a clinical entity that results from prolonged and chronic stress leading to physical, emotional, and mental exhaustion [20]. Occupational burnout takes place when a person is incapable to meet constant job demand, feels emotionally drained, and overwhelmed. In the late 1960s, the concept of burnout in health care has emerged to describe the psychological and emotional stress among HCPs, reflecting the job-related stress in health care facilities [21]. Several factors act as driving forces of burnout, some of which are, job-related stress, high workload, unhealthy work environment as well as insufficient organizational support [17, 22].

In addition, burnout can lead to deterioration in the level of job satisfaction among HCPs. Physicians’ and nurses’ burnout may result in negative organizational outcomes such as absenteeism, less patient satisfaction, and low quality of care; thus, occupational burnout can be recognized as an occupational disease impacting the health of HCPs and their productivity as well [23, 24]. Job satisfaction is a complex concept with a variety of definitions, nevertheless, it can be briefed as an individualized positive feeling and attitude toward a job [18, 25]. A recent systematic review conducted by Domagała et al. has revealed that characteristics of a workplace, work conditions, professional development, management quality, and team support were significant factors for physicians’ job satisfaction [26]. Besides, a study that was conducted by Dimitriu et al. during the COVID-19 pandemic, revealed that occupational burnout was found in 76% of physicians included in their study, reflecting an alerting level compared to prior studies [27]. Moreover, literature has shown that the COVID-19 pandemic has significantly impacted the psychological well-being of HCPs [8, 28, 29], making them vulnerable to suffer from burnout as well. Preliminary results from a study that was conducted in October 2020 by the British Medical Association found that 43.7% of participating doctors were suffering from symptoms related to anxiety, depression, emotional distress, and burnout during their medical practice amid the current pandemic [30]. In the same study, around 59.0% of doctors also reported higher-than-normal levels of occupational exhaustion and fatigue during the pandemic.

Physicians as a part of HCPs play an essential role in the battle against the COVID-19 pandemic, with their priceless and immense sacrifices provided every day in this long battle alongside other HCPs. Considering the fact that this pandemic has forced many changes in different aspects of healthcare services delivery and workload, this is expected to have emotional, physical, and psychological impacts on physicians. During the COVID-19 pandemic, medical practice has been also forced to change due to multiple preventative measures that were implemented worldwide to control the spread of the disease. In Jordan, these actions and measures called for the readjustment and reorganization of activities of HCPs to face repercussions of the pandemic. Considering the previously described pandemic-related impacts on physicians that were addressed in various studies, the possible pandemic effects on physicians’ day-to-day work, the escalated COVID-19 morbidity and mortality in Jordan, and the plethora of emotions and distress that may accompany the pandemic crisis, the purpose of our study was determined to investigate burnout and job satisfaction among physicians in Jordan as a subpopulation of HCPs due to scarcity of literature that addressed this issue among physicians in the region. Specific aims of our study were (i) To assess the prevalence of burnout, levels of job satisfaction, as well as their predictors among practicing physicians in Jordan during the COVID-19 pandemic, and (ii) To explore physicians’ experiences and perceptions during their daily clinical practice amid the pandemic crisis as well as their opinions regarding the imposed measures at their workplaces.

## Methodology

### Study design

**Fig. 1:**
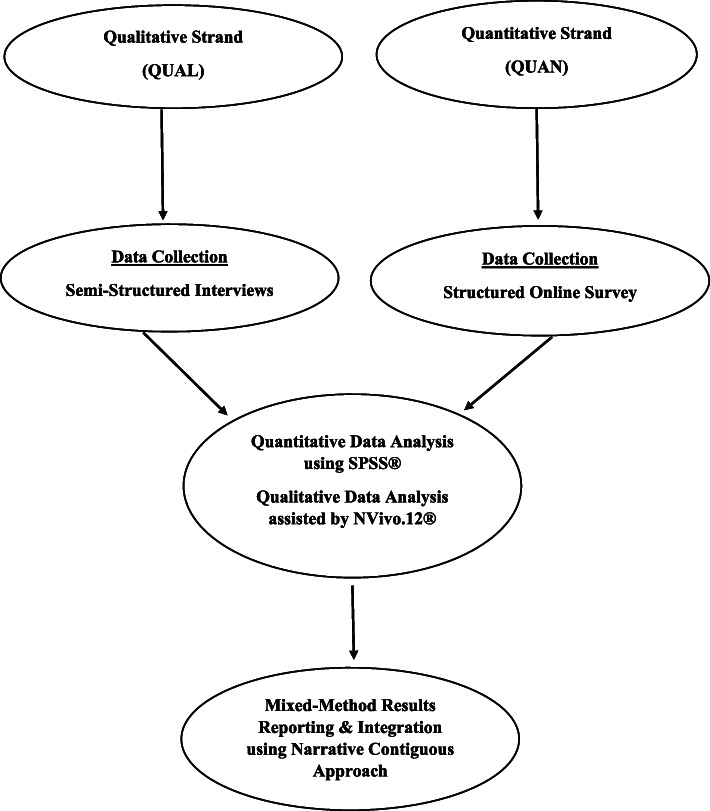
Flowchart of Convergent Parallel Mixed-Method Design Implemented in our present study

Due to complex interactions between occupational burnout and job satisfaction, and in order to gain a comprehensive and in-depth overview of this topic narratively and numerically, a convergent parallel multi-strand mixed-method design was implemented [31, 32]. In the current study, the research aims were addressed using both qualitative and quantitative methods; thus, achieving a comprehensive interpretation of data based on the power of methods triangulation [33]. A mixed-method approach is characterized by the integration that happens between qualitative and quantitative methods at single or multiple steps of research [34]. In our study, quantitative (QUAN) and qualitative (QUAL) strands were implemented concurrently with equal weight; therefore, given the notation QUAN+QUAL [33]. Both quantitative and qualitative data collection methods were utilized. Data collection for both strands occurred in parallel, i.e., in the same timeframe approximately. Then, data were analyzed simultaneously and independently after completing data collection for both strands. Finally, results from both mixed-method strands were integrated to look for convergence, divergence, and expansion [34]. See Fig. 1.

### Study setting and study population

Our study was conducted in Jordan, a middle-income country located in the Eastern Mediterranean Region (EMR) with a total population of 10.8 million [35]. Our study population was physicians of various specialties and from different institutional representatives of the health sector in Jordan, including the Jordanian Ministry of Health (JMOH), Jordanian Royal Medical Services (JRMS), University Hospitals (UHs), Non-Governmental Organizations (NGOs), and Private Hospitals (PHs).

According to the acting president of the Jordanian Medical Association (JMA), a total of 24,000 physicians are registered at JMA, of whom 16,000 physicians practice their jobs in Jordan [36].

### Quantitative data collection and sampling: QUAN Strand

In this strand, a structured cross-sectional questionnaire was developed (See Additional file 1). The survey questionnaire comprised three sections with a total of 32 questions. The first section included 17 questions distributed as the following: eight questions about sociodemographic profile including age, gender, marital status, smoking status, number of household members, type of household (rented vs owned), geographical region of residence, average monthly salary (in Jordanian Dinars), and nine questions about participants’ work profile including, Job-status (full-time vs part-time), professional classification (general practitioner, resident, specialist, consultant), Speciality discipline (medical vs surgical), average working hours per week, night duties per week, healthcare institution, sufficient access to personal protective equipment (PPE), COVID-19 related financial incentives, and the participants’ COVID-19 status. The sociodemographic and work-related factors were developed by the research team based on the possible factors that may have a role in job-related burnout and job satisfaction during the pandemic crisis, as well as from relevant published literature [17, 26, 37–41].

Our survey’s second section adopted the *10-item Burnout Measure-Short version (BMS)* by Malach-Pines [42]. The 10-item BMS is an easy-to-administer brief instrument that comprises a series of 10 questions that assess an individual’s level of physical exhaustion, emotional exhaustion as well as mental exhaustion based on the main constructs of the concept of burnout. In the 10-item BMS tool, participants were asked to report to what extent their jobs make them feel tired, disappointed, hopeless, trapped, depressed, physically exhausted/sickly, worthless/sense of failure, difficulties in sleeping, and lastly if no longer willing to do the job. Each item is evaluated using 7-point Likert-type responses (from 1 = never to 7 = always). Based on the response value for each item, the total response points (for all of 10 items) fall between 10 and 70. To calculate the overall burnout score for each participant, the result of summing up each participant’s response values is divided by 10. Thus, the overall burnout score will be in the range of 1 to 7. According to Malach-Pines, an overall score ≥ 4 indicates an established state of burnout. Therefore, the overall burnout score categorizes participants into two groups; those who have burnout (overall score ≥ 4), and those who are not likely to have burnout (overall score < 4). The 10-item BMS was validated on a sample of Arabs (including an occupational sample from a healthcare setting) and has shown satisfactory psychometric properties in terms of internal consistency and reliability with Cronbach α = 0.85 [42]. In our present study, the Cronbach α for the 10-item BMS was 0.91.

The third section of our survey has adopted the *5-Item Short Index of Job Satisfaction (SIJS)*. The 5-item SIJS is a psychometric scale that includes five items about self-reported job satisfaction. The original 18-item Index of Job Satisfaction (IJS) was developed by Brayfield and Rothe [43]. The shorter version of it, the so-called 5-item SIJS has been previously proposed and validated as well [44]. The 5-item SIJS includes a series of five questions about self-reported states of self-satisfaction with a job, enthusiasm about the job, real enjoyment in work, feeling unpleasant in a job, and feeling that a day in work will never come to an end. Responses are scored on a 5-point Likert scale (from 1 = strongly disagree to 5 = strongly agree) with two items are reversely scored; thus, the overall Job satisfaction score for each participant falls between 5 and 25 with no cut-off score i.e., higher scores indicate more levels of job satisfaction [45]. The 5-item SIJS was reported to have a satisfactory internal consistency with Cronbach α in the range of 0.82–0.89 [45]. Besides, the 5-item SIJS has shown to have a measurement invariance across countries and gender. In our study, the Cronbach α for the 5-item SIJS scale was 0.81.

To limit physical contact with physicians due to the current COVID-19 pandemic and to eliminate the geographical boundaries aiming to reach participants from different Jordanian governorates, the cross-sectional survey was developed as a web-based survey using Google Form® (a cloud-based survey tool) and was distributed to physicians in Jordan through social media platforms including Facebook®, WhatsApp®, Facebook messenger®, and LinkedIn®. Data collection was carried out in the period October 29 – November 12, 2020.

Participants were encouraged to further disseminate the survey’s link to their workmates who are physicians; thus, employing a snowball convenience sampling strategy. The inclusion criterion for participation was being a practicing physician in Jordan during the COVID-19 pandemic. Participation was voluntary and participants who were interested to fill out the survey questionnaire have provided written informed consent electronically. Regarding the sample size for the QUAN strand, based on the declared number of practicing physicians in Jordan which is 16,000 [36], and by using Open Source Epidemiologic Statistics for Public Health Version 3.01 [46], a sample size of at least 376 participants was required, with 95% confidence level, 50% response distribution and 5% margin of error. To evaluate the clarity and understandability of our survey questions, the questionnaire was piloted on 35 physicians. No amendments were needed based on the pilot test. The pilot responses were excluded from our analysis.

### Qualitative data collection and sampling: QUAL Strand

This strand utilized semi-structured individual interviews as a tool for data collection using an interview guide [47, 48] (See Additional file 2). Given the unfolding situation of the COVID-19 pandemic and its control measures, and due to difficulty in conducting face-to-face interviews from a logistic perspective, the interviews were conducted via telephone in the period November 8–November 20, 2020. The conceptual framework of our QUAL strand was based on two theoretical models, namely: (i) Herzberg’s Two-Factor Theory of Motivation (also known as Motivation-Hygiene Theory) [49] and, (ii) Job Demands–Resources Model [50].

**Fig. 2:**
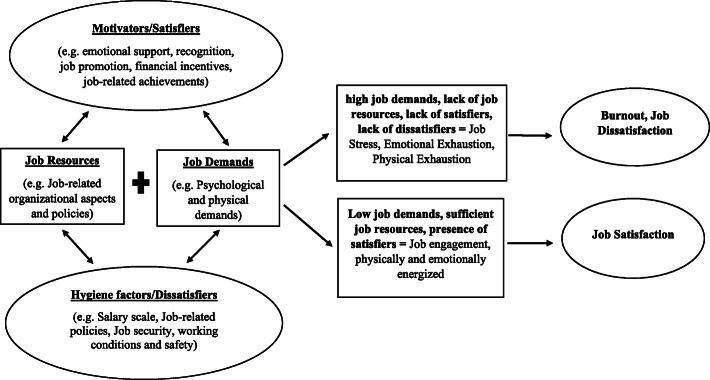
Conceptual Framework of the Qualitative Strand, based on Herzberg’s Two-Factor Theory of Motivation and The Job Demands–Resources Model

Herzberg (1966) [49] had reported that job satisfaction is a result of interaction between two categories of job factors. The first category relates to *hygiene factors* which are essential for the existence of motivation at the workplace. The absence of hygiene factors will lead to dissatisfaction; however, their presence may not necessarily result in job satisfaction. The other category relates to *motivational factors* that motivate an individual for better performance; thus, leading to job satisfaction if present and to job dissatisfaction if not [49]. Besides, the Job Demands-Resources model provides a framework that helps to understand the interactions between job characteristics and their impacts on the well-being of employees [51]. The interaction between *job demands* (e.g. physical energy and skills needed to successfully perform a job) and *job resources* (e.g. factors that facilitate doing a job, motivate the employee and reduce the job demands) determine the level of occupational stress and burnout [20, 50]. We adopted some concepts from the abovementioned theoretical models and developed the conceptual framework of our study’s QUAL strand. See Fig. 2.

Participants in the individual interviews were recruited purposively using maximum variation sampling (different healthcare institutions, age, gender, marital status, specialties, and professional classifications) to achieve diversity in the participants’ views, experiences, and opinions [33]. Besides, participants who enrolled in the individual interviews did not participate in the cross-sectional survey to ensure having two parallel independent datasets and to provide diverse perspectives from each mixed-method strand [33].

Unlike the case of the QUAN strand, no predetermined sample size was needed as we used the principle of saturation to reach a proper sample size for the QUAL strand i.e. when no more new information or insights can be collected by conducting further interviews [47, 48, 52]. The interviews were conducted by the first author (male, medical doctor, researcher, and board-certified ophthalmologist) and the second author (male, medical doctor, public health specialist with experience in psychological research). Also, various web-based sessions were conducted between the first and second authors regarding qualitative methodology (interviews and analysis) prior to data collection and during data analysis and reporting. The interviews were audio-recorded, saved, and encrypted to ensure the anonymity and confidentiality of participants. Each participant provided informed consent to participate in the interview and to record it as well. A total of 11 physicians were interviewed, and the average duration of an interview was approximately 18 min.

### Data analyses and integration technique

For the QUAN strand, completed questionnaires were extracted from Google Form® then were entered into Statistical Package for Social Sciences, SPSS® version 27.0 for Windows (IBM Corp. Version 27.0. Armonk, NY) for quality check and analysis. Descriptive and summary statistics were performed for categorical and numerical data. Frequencies, proportions, means, and range were presented as appropriate. Forward stepwise multivariable logistic regression was employed to predict the association between burnout (binary outcome variable) and multiple explanatory variables including sociodemographic and work-related factors. The relationship between the outcome variable and each explanatory variable was firstly assessed using univariable analysis. Any explanatory variable with a *p*-value < 0.25 in univariable analysis, was considered a candidate for the forward stepwise regression [53].

Regarding Job satisfaction score, which is a scale outcome variable, forward stepwise multiple linear regression was utilized to test the association between satisfaction score and sociodemographic and work factors. Also, explanatory variables with a *p*-value < 0.25 in univariable analysis, were considered candidates for the multiple linear regression analysis [53].

All assumptions of linear regression were checked and met, including normality of outcome variable and linearity between the outcome variable and explanatory variables. Homoscedasticity was assessed by the Bresch-Pagan test, which revealed a *p*-value =0.124 in our study; thus, indicating homoscedasticity. Besides, to ensure the absence of multicollinearity in both regression analyses, cut-off values of variance inflation factor < 10 and tolerance > 0.1 were used. The confidence level was set at 95% and a *p*-value < 0.05 was implemented for statistical significance.

For the QUAL strand, audio records of telephone interviews were transcribed verbatim. Then, transcribed data were analyzed inductively using thematic analysis as suggested by Braun and Clarke [54]. This involved familiarization with data, developing initial codes, merging codes into themes, reviewing themes, defining and naming themes, and lastly reporting themes. Qualitative data management was assisted by using a qualitative data analysis software NVivo® for Windows (QSR International, release 1.3, 2020). Data coding and qualitative data analysis were conducted by the first and second authors. Any discrepancies in coding were discussed by the research team and resolved by reaching a consensus.

For reporting and integration of our mixed-method results, a *narrative contiguous approach* was implemented, that is, reporting results of the QUAN strand followed by results of the QUAL strand in different subsections [31].

### Ethical considerations

The protocol of this mixed-method study was reviewed and approved by the ethics committee of the Faculty of Medicine at Mutah University in Jordan (Reference Number: 1112020). In addition, this study was conducted conforming to the Declaration of Helsinki. The cross-sectional survey ensured the confidentiality and anonymity of the study participants. Moreover, participation in both strands was voluntary. Regarding the qualitative strand, audio-recorded interviews as well as the transcribed data were encrypted and kept securely. Moreover, electronic written informed consent was obtained from all participants included in both strands.

## Results interpretation and integration

### QUAN Strand: cross-sectional survey

In the QUAN strand, a total of 1037 physicians participated in the cross-sectional survey. 64 responses were excluded because participants did not provide informed consent or did not meet the inclusion criterion. Thus, the final number of responses included in the quantitative analysis was 973. Regarding the sociodemographic profile of respondents, the mean age was 34.6 (SD: 9.9) with 68.4% being within the age group (24–34) years. Most physicians were males (*n* = 679, 69.8%), married (*n* = 585, 60.1%), living with household members of one to three persons (*n* = 461, 47.4%), own their flats or houses (*n* = 663, 68.1%), living in central governorates (*n* = 764, 78.5%), non-smokers (*n* = 569, 58.5%), and of income category 700–1400 Jordanian Dinars (*n* = 412,42.3%). The respondents’ work profile showed that most physicians were classified as resident doctors (*n* = 444, 45.6%), from medical specialties/departments (*n* = 559, 57.5%), working at JMOH hospitals (*n* = 312, 32.1%), involved in a full-time job (*n* = 833, 85.6%), working more than 48 h per week (*n* = 380,39.1%), doing 1–3 night shifts per week (*n* = 547, 56.2%), had sufficient access to PPE at workplace (*n* = 532, 54.7%), did not receive any financial incentives while working during pandemic crisis (*n* = 900, 92.5%). Besides, 154 physicians (15.8%) were positively tested for SARS-CoV-2. Tables 1 and 2 show more descriptive details about the sociodemographic and work profile of respondents in the cross-sectional survey, including more details about variables’ categorization.

**Table 1:** Socio-Demographic Profile of Physicians who Participated in the Cross-Sectional Survey, Total Number of Participants = 973

Variables	N (%)
**Age (Mean ± SD, Range)**	34.6, 9.9, 24–77
(24–34)	666 (68.4)
(35–45)	193 (19.8)
(46–56)	53 (5.4)
(57–77)	61 (6.3)
**Gender**	
Male	679 (69.8)
Female	294 (30.2)
**Marital Status**	
Single (never married, divorced, widowed)	388 (39.9)
Married	585 (60.1)
**Household Members**	
1–3 Persons	461 (47.4)
4–6 Persons	379 (39.0)
> 6 Persons	133 (13.7)
**Residence Place**	
Rented	310 (31.9)
Owned	663 (68.1)
**Geographical Region of Residence**	
Northern Governorates	167 (17.2)
Central Governorates	764 (78.5)
Southern Governorates	42 (4.3)
**Smoking Status**	
Non-Smoker	569 (58.5)
Smoker	404 (41.5)

**Table 2:** Work Profile of Physicians who Participated in the Cross-Sectional Survey, Total Number of Participants = 973^a^

Variables	N (%)
**Speciality Discipline**	
Medical	559 (57.5)
Surgical	414 (42.5)
**Professional Classification**	
General Practitioner	148 (15.2)
Resident	444 (45.6)
Specialist	241 (24.8)
Consultant	140 (14.4)
**Workplace**	
JMOH	312 (32.1)
JRMS	252 (25.9)
PHs	279 (28.7)
NGOs	38 (3.9)
UHs	92 (9.5)
**Job Status**	
Full-Time	833 (85.6)
Part-Time	140 (14.4)
**Average Monthly Salary**	
< 700 JDs	372 (38.2)
700–1400 JDs	412 (42.3)
> 1400 JDs	189 (19.4)
**Duty Hours/Week**	
< 40	221 (22.7)
40–48	372 (38.2)
> 48	380 (39.1)
**Night shifts/Week**	
None	300 (30.8)
1–3	547 (56.2)
> 3	126 (12.9)
**Sufficient PPE at Workplace**	
Yes	532 (54.7)
No	441 (45.3)
**COVID-19 Related Financial Incentives**	
Yes	73 (7.5)
No	900 (92.5)
**Tested Positive for SARS-COV-2**	
Yes	154 (15.8)
No	819 (84.2)

^a^*JMOH* Jordanian Ministry of Health, *JRMS* Jordanian Royal Medical Services, *NGOs* Non-Governmental Organizations, *PHs* Private Hospitals, *UHs* University Hospitals, *PPE* Personal Protective Equipment, *JDs* Jordanian Dinars (official currency of Jordan, 1 JD = 1.4 U.S. Dollars)

Based on the burnout score’s cut-off point (≥ 4), and out of the total sample (*n* = 973, 100%), 376 male physicians and 185 female physicians were found to suffer from an established state of burnout; thus, the overall prevalence of burnout among physicians in our study was 57.7%.

The logistic regression model revealed several explanatory variables that acted as potential predictors of occupational burnout among physicians, including being *a female physician* was associated with statistically significant higher odds of suffering from burnout (aOR = 1.445; *p* = 0.030; 95% CI: 1.035, 2.017) compared to males as a reference category. In addition, *working at JMOH and JRMS hospitals* was found to be associated with higher possibilities of suffering from burnout with (aOR = 2.377; *p* = 0.002; 95% CI: 1.359, 4.155) and (aOR = 2.258; *p* = 0.006; 95% CI: 1.269, 4.019), respectively. Physicians who worked *more than 48 h/week* (aOR = 1.585; *p* = 0.043; 95% CI: 1.016, 2.474), had *one to three night shifts/week* (aOR = 2.078; *p* < 0.001; 95% CI: 1.438, 3.003), had *more than three night shifts/week* (aOR = 2.101; *p* = 0.007; 95% CI: 1.230, 3.590), with *no sufficient access to PPE at workplace* (aOR = 2.754; *p* < 0.001; 95% CI: 2.038, 3.723), and with *confirmed COVID-19 status* (aOR = 1.626; *p* = 0.026; 95% CI: 1.061, 2.491) were found to act as statistically significant predictors for suffering from occupational burnout compared to their corresponding reference categories. See Table 3 for a more detailed representation of the logistic regression results.

**Table 3:** Results of Multivariable Logistic Regression for the association between Burnout and Sociodemographic/Work factors (final model^a^)

Predictors	Adjusted Odds Ratio	95% Confidence Interval		*P*-value
		Lower	Upper	
Age	0.982	0.963	1.002	0.81
Gender				
Male (Reference)				
Female	1.445	1.035	2.017	**0.030**
Workplace				
UHs (Reference)				
JMOH	2.377	1.359	4.155	**0.002**
JRMS	2.258	1.269	4.019	**0.006**
PHs	1.276	0.731	2.230	0.391
NGOs	1.805	0.775	4.205	0.171
Monthly Income				
> 1400 JDs (Reference)				
< 700 JDs	1.867	1.116	3.123	**0.017**
700–1400 JDs	1.347	0.852	2.129	0.203
Duty Hours/Week				
< 40 (Reference)				
40–48	0.887	0.601	1.311	0.548
> 48	1.585	1.016	2.474	**0.043**
Night Shifts/Week				
None (Reference)				
1–3	2.078	1.438	3.003	**< 0.001**
> 3	2.101	1.230	3.590	**0.007**
Sufficient PPE				
Yes (Reference)				
No	2.754	2.038	3.723	**< 0.001**
SARS-CoV-2 Infection				
No (Reference)				
Yes	1.626	1.061	2.491	**0.026**

^a^Statistically significant *P* values at *P* < 0.05 are in **Bold**. Model’s Nagelkerke *R*^*2*^ = 0.274; Hosmer and Lameshow test: *X*^*2*^ (8) = 9.001, *P*-value = 0.342; *JMOH* Jordanian Ministry of Health, *JRMS* Jordanian Royal Medical Services, *NGOs* Non-Governmental Organizations, *PHs* Private Hospitals, *UHs* University Hospitals, *PPE* Personal Protective Equipment, *JDs* Jordanian Dinars (official currency of Jordan, 1 JD = 1.4 U.S. Dollars)

As described in the methodology, the job satisfaction score using the 5-item SIJS scale has an overall value between 5 and 25 with no cut-off scores; thus, the higher the score, the more job satisfaction. The mean score (SD) of job satisfaction among male physicians and female physicians was 15.3 (SD: 3.98) and 15.5 (SD: 3.80), respectively. However, this difference in job satisfaction scores between different gender was statistically not significant (*p* = 0.562). The multiple linear regression model revealed that with an increase in *age*, there is a statistically significant increase in job satisfaction (β = 0.066; *p* = 0.017; 95% CI: 0.005, 0.047). Expectedly, the level of *burnout* was a significant predictor of job satisfaction, that is, having higher scores of burnout leads to lower levels of job satisfaction (β = − 0.600; *p* < 0.001; 95% CI: − 2.225, − 1.888). In addition, being *a general practitioner or a specialist* predicted lower levels of job satisfaction (β = − 0.092; *p* < 0.001; 95% CI: − 1.597, − 0.472) and (β = − 0.070; *p* = 0.006; 95% CI: − 1.087, − 0.180), respectively, compared to reference category. Moreover, working at *JMOH hospitals* (β = − 0.147; *p* < 0.001; 95% CI: − 1.678, − 0.793), working at *JRMS hospitals* (β = − 0.095; *p* < 0.001; 95% CI: − 1.346, − 0.359) having a monthly *income less than 700 Jordanian Dinars* (β = − 0.080; *p* = 0.008; 95% CI: − 1.115, − 0.170) predicted lower levels of job satisfaction compared to their reference categories. See Table 4 for detailed results.

**Table 4:** Results of Multivariable Linear Regression for the association between Job Satisfaction Score and Sociodemographic/Work factors (final model^a^)

Predictors	β coefficient	*P*-value	95% Confidence Interval	
			Lower	Upper
Age	0.066	**0.017**	0.005	0.047
Burnout	−0.600	**< 0.001**	−2.225	−1.888
Smoking Status				
Non-Smoker (Reference)				
Smoker	−0.054	**0.021**	−0.795	−0.064
Workplace				
UHs (Reference)				
JMOH	−0.147	**< 0.001**	−1.678	−0.793
JRMS	−0.095	**< 0.001**	−1.346	−0.359
Professional Classification				
Consultant (Reference)				
General Practitioner	−0.092	**< 0.001**	−1.597	− 0.472
Specialist	−0.070	**0.006**	−1.087	−0.180
Monthly Income				
> 1400 JDs (Reference)				
< 700 JDs	−0.080	**0.008**	−1.115	−0.170

^a^ Statistically significant *P* values at *P* < 0.05 are in **Bold,** model’s adjusted *R*^*2*^ = 0.467; other variable categories and subcategories were not chosen by the stepwise forward regression algorithm. *JMOH* Jordanian Ministry of Health, *JRMS* Jordanian Royal Medical Services, *UHs* University Hospitals, *JDs* Jordanian Dinars (official currency of Jordan, 1 JD = 1.4 U.S. Dollars)

### QUAL Strand: individual interviews

A total of 11 physicians were interviewed. Table 5 describes the interviewees’ characteristics. In this strand, four main themes have emerged from the qualitative thematic analysis, namely (i) Work-induced psychological distress during the COVID-19 pandemic, (ii) Decision-driven satisfactory and dissatisfactory experiences, (iii) Impact of the pandemic on doctor-patient communication and professional skills, and lastly, (iv) The economic impacts of the pandemic crisis and lockdown.

**Table 5:** Characteristics of Physicians who participated in the Semi-Structured Individual Interviews

No. of Interviewees	*n* = 11
**Age (mean, range)**	36.2, 28–56
**Marital Status**	Single (*n =* 3), Married (*n =* 8)
**Workplace**	Jordanian Royal Medical Services (*n =* 3), Jordanian Ministry of Health (*n =* 3), Private Hospitals/Clinics (*n =* 5)
**Classification**	General Practitioner (*n* = 2), Resident (*n =* 5), Specialist (*n =* 2), Consultant (*n =* 2)
**Speciality Discipline**	Orthopedics (*n =* 1), Pediatrics (*n* = 1), General Practice (*n =* 2), Ophthalmology (*n =* 1), Internal Medicine (*n* = 2), Neurosurgery (*n =* 1), Intensive Care (*n =* 1), Otorhinolaryngology (*n =* 1), Radiology (*n* = 1)

#### Theme 1: work-induced psychological distress during the COVID-19 pandemic

During the current pandemic, physicians were exposed to higher levels of workload imposing a significant burden on their physical and psychological well-being. Most of the physicians who participated in the interviews reported fear and anxiety related to their medical practice amid the pandemic crisis. The fear and anxiety were mainly a result of their worries about catching the virus and transmitting it to their beloved family members. This could be expected to act as a contributing factor to their emotional and mental exhaustion; leaving them more vulnerable to suffer from burnout.

A physician said:

*“There is a significant distress during my work amid the COVID-19 pandemic due to being fearful of transmitting the infection to my family after returning to home ”* (Participant #1, Male, Private Sector)

Also, another physician added the following:

*“After a day of clinical practice, the most anxious thing is being afraid of being infected as well as transmitting the virus to my family and elderly household members”* (Participant #2, Male, Private Sector)

In addition, the rising COVID-19 morbidity and mortality in Jordan was also another factor that afflicted the psychological status of physicians:

*“ … I feel a significant degree of psychological distress due to the increasing number of COVID-19 cases and deaths in our community and during my daily practice … ”* (Participant #6, Male, JMOH)

Alongside distress, an intensivist physician said:

*“As an intensivist, I finish my duty with deep sadness considering the levels of suffering I observe among COVID-19 patients at the critical care unit accompanied by the rising number of deaths … ”* (Participant #5, Male, Private Sector)

#### Theme 2: decision-driven satisfactory and dissatisfactory experiences

In the interviews, physicians have reported numerous satisfactory and dissatisfactory job experiences during their clinical practice amidst the COVID-19 pandemic. The experiences were attributed to various pandemic-related decisions at institutional, sectoral as well as national levels, such as decisions on PPE supply, health workforce, and the COVID-19 control measures. These experiences whether satisfactory or dissatisfactory could have a profound impact on the level of job satisfaction as well as the emotional and physical well-being of physicians. A physician has said:

*“I think that the protocols implemented at my workplace during the COVID-19 pandemic were satisfactory to me from a practice perspective, but they could have been better than that”* (Participant #1, Male, Private Sector)

On the other side, a physician added:

*“I noticed a lack of experience as well as many conflicting decisions among the managerial panel at my workplace, especially decisions regarding health workforce capacity and personal protective equipment supply. More health care professionals, especially nurses and doctors are needed at critical care units during the COVID-19 crisis”* (Participant #5, Male, Private Sector)

Other physicians have expressed the impact of imposed measures and decisions on the level of job satisfaction:

*“In my opinion, reluctant and irrational decisions imposed by the pandemic taskforce, false information in the society as well as the lack of transparency in the governmental speech, were all dissatisfactory to me in terms of my personal and professional life aspects ”* (Participant #10, Male, Private Sector)

*“I felt a swing between satisfaction and dissatisfaction during my duty, depending on the availability of personal protective equipment at the workplace and the effectiveness of patients triaging system during my shift”* (Participant #11, Female, JRMS)

Moreover, collaborative efforts at the workplace were described as a motivational factor for better and more efficient engagement in work:

*“As a healthcare professional, I feel proud of my sacrifices during the COVID-19 pandemic … the spirit of collaborative teamwork has motivated me during my work amid this crisis”*(Participant #8, Female, JRMS)

Acknowledgments and financial incentives are considered important factors for motivating employees to be more productive. A physician has mentioned that reliable acknowledgment is also needed to encourage healthcare workers to provide more, and to compensate them as well:

*“There should be a reliable acknowledgment to healthcare workers during the pandemic crisis, emotionally and financially, considering the sacrifices we provide every day”* (Participant #6, Male, JMOH)

As found in the QUAN strand, various explanatory predictors have been identified as risk factors for occupational burnout among the participating physicians. Some of these predictors such as workplace environment, workload, and the availability of PPE were also reported by physicians who participated in the interviews. Besides, law enforcement of mask-wearing and pandemic-related managerial decisions were expressed to exhibit a significant impact on physicians and their practice. For example, some physicians have expressed positive opinions regarding some pandemic-related decisions on COVID-19 precautionary measures as well as the extent of non-emergent healthcare services at their workplaces:

*“In my opinion, obligatory mask-wearing was amongst the most vital decisions which were legally enforced by the government recently to limit the viral spread in my workplace environment, and this has positively impacted my work conditions and medical practice”* (Participant #1, Male, Private Sector)

*“I believe that many good decisions were executed by decision-makers, such as limiting non-emergent healthcare services to reduce the possibility of COVID-19 community spread and reducing the risk of COVID-19 spread at the workplace ”* (Participant #8, Female, JRMS)

On contrary, other physicians believed that many pandemic-related decisions at workplaces were not executed properly, in terms of lack of sufficient access to PPE, higher workload (day and night shifts), and limited health workforce:

*“ … I did not notice any escalation of precautionary measures at my workplace except for more monitoring of mask-wearing among healthcare workers and visitors … ”* (Participant #3, Male, JMOH)

Moreover, Physicians have pointed to the emotional and physical fatigue caused by higher workload which has negatively impacted their satisfaction:

*“Overall, I am not satisfied with the working conditions at my workplace during the COVID-19 pandemic. Lack of a sufficient number of healthcare workers, as well as the lack of a unified protocol to be followed in dealing with suspected cases, have imposed more workload and distress, respectively”* (Participant #3, Male, JMOH)

*“During the COVID-19 pandemic, I am not satisfied with my working conditions, lack of any form of emotional support or even safety incentives, coupled with a low number of healthcare workers, have all led to an increased workload on me and my colleagues”* (Participant #2, Female, JMOH)

#### Theme 3: impacts of the pandemic on doctor-patient communication and professional skills

During the current pandemic, physicians have experienced a change in the classical doctor-patient communication during the pandemic, and this was an expansion of the predicting factors of job satisfaction that could exhibit a bidirectional effect from the physicians’ perspectives. The COVID-19 pandemic has pushed various sectors to work and communicate remotely through virtual means:

*“Using digital platforms for consultation and patient follow-up is considered one of the newly emerged communication methods in healthcare practice in Jordan. Healthcare professionals especially doctors were obliged to learn the digital health skills and effectively utilize them during the pandemic crisis”*

(Participant #1, Male, Private Sector)

On the other hand, a physician has expressed an increase in the level of interaction with patients:

*“ … Being a member of the epidemiological surveillance team, my communication with people has significantly increased during the COVID-19 pandemic … ”* (Participant #4, Female, JMOH)

Physicians had also worrisome concerns about their communication with patients and other professional skills:

*“As a pediatrician, there was a noticeable difficulty in communicating with families and following up my patients especially the newly discharged neonates and infants using the telephone. There was a lack of clinical and milestone examinations as this needs in-person attendance to the clinic which was impacted by the lockdown measures”* (Participant #2, Male, Private Sector)

Surgeons seemed to be also worried about their surgical skills, two surgeons said:

*“I was worried about my surgical skills during the long period of nationwide lockdown in Jordan and any further lockdowns in the future”* (Participant #9, Male, Private Sector)

Another surgeon added:

*“Returning to surgical practice after a long lockdown was challenging, because surgeons rely on daily surgical skills and practice. I believe that dealing with surgical operations using a guided step-by-step approach helped me to pass through the challenge of not performing surgical procedures for few months during the lockdown in Jordan”* (Participant #1, Male, Private Sector)

#### Theme 4: the economic impacts of the pandemic crisis and lockdown

Salary is considered an important factor for job satisfaction. In our cross-sectional survey, early-career physicians who had an average monthly income below 700 JDs were found to have lower job satisfaction. No one would deny that the COVID-19 pandemic has negatively impacted the world’s economy. This was partially a result of the lockdown/curfew policy imposed by many countries including Jordan, alongside other control measures. Physicians as part of the society were also vulnerable to suffer from pandemic-induced economic impacts.

A Physician said:

*“The lockdown policy has severely impacted individuals who do not have a stable monthly income such as daily workers. Despite that, there was a sort of community-governmental partnership at the early stages of lockdown in Jordan for supporting individuals with unstable earnings, but this has gradually decreased by the time”* (Participant #2, Male, Private Sector)

Another physician added the following:

*“From an economic perspective, I was worried about my financial stability due to the lockdown and the governmental decisions that allowed the managerial panel of the healthcare sector as well as other sectors in Jordan to deduct from their employees' salaries as per the declared defense law in Jordan”* (Participant #4, Female, JMOH)

Some physicians in the private sector were also impacted financially due to the lockdown policy in Jordan:

*“As an owner of a private clinic, and during the pandemic crisis, my financial status has deteriorated due to the lockdown and even after the gradual return to functioning economy. People were hesitant to visit private healthcare institutions due to their financial crisis during the pandemic”* (Participant #10, Male, Private Sector)

*“I think that the pandemic crisis has already impacted everyone in society economically, even healthcare workers. I have witnessed many cases in which salaries of healthcare workers including medical doctors were partially deducted, especially in the private sector. This has led to negative psychological impacts on us”* (Participant #5, Male, Private Sector)

Despite that many physicians reported higher financial burden due to the pandemic, some physicians have not been financially impacted, considering the healthcare institutions where they work:

*“This pandemic did not afflict my salary as I work at a governmental healthcare institution; thus, having a stable monthly income”* (Participant #3, Male, JMOH)

*“During the lockdown in Jordan, I did not feel that there was a direct impact on my monthly income as I have a stable salary every month … however, I noticed that prices of many consumer goods have been raised during the lockdown and curfew”* (Participant #7, Female, JRMS)

Also, another physician stated the following:

*“My salary was not affected by the pandemic crisis because I work in a military healthcare institution, therefore, I have a stable monthly income from the government”* (Participant #11, Female, JRMS)

## Discussion

Our present study sheds the light on an important, yet under-investigated topic amid the COVID-19 pandemic. Burnout is a major global issue that afflicts HCPs of various specialties and disciplines. In low and middle-income countries, burnout was reported to be in the range of 43–48% among HCPs before the current pandemic [55]. In the Middle East, where Jordan is located, a recent systematic review that assessed the burden of burnout among HCPs, including physicians and nurses, found that prevalence estimates of burnout were in the range of 40–60% [56]. However, burnout has not received much attention in the Middle East despite the existence of well-identified stressors in healthcare settings, considering the paucity of resources in many EMR countries, civil wars in the region, and the refugee crisis [56].

The prevalence of burnout in our study was about 57.7% and several factors were identified to be positively associated with burnout among physicians during the pandemic crisis. These factors included female gender, workplace, salary, workload (in terms of duty hours and night shifts), access to PPE, and lastly a physician’s COVID-19 status. Complex interactions between these factors could have exhibited either direct or indirect effects on physicians’ physical, emotional, and psychological wellbeing; thus, leading to a state of burnout.

The rate of burnout in our study is closely similar to the rate reported by Morgantini et al. in their cross-sectional study that involved 2707 HCPs from 60 countries (51.4%) [17]. Also, our study found that the lack of sufficient supply of PPE acted as a predisposing factor for burnout during clinical practice amid the pandemic, and this finding is consistent with Morgantini et al. study that reported a sufficient supply of PPE to act as a protective factor against burnout during COVID-19 pandemic.

In addition, most physicians in our qualitative strand expressed that availability and sufficient access to PPE was a distressing concern to them at their workplaces. Preparedness at individual and institutional levels is crucial in the fight against the current pandemic [57]. In a study conducted by Suleiman et al. among frontline physicians in Jordan, only 18.5% of participants had reported sufficient access to PPE [57]. Additionally, qualitative findings from an embedded mixed-method study that was conducted in Malaysia during the COVID-19 pandemic pointed to various factors as a source for burnout among healthcare workers, including factors related to workload (e.g., long duty hours, working with high precuations, insufficient manpower), Pandemic course uncertainity and unpredictability of events (e.g., frequent changes of operation protocols and roles, unprecedented changes in personal plans), a challenging work-family balance (e.g., fear and anxiety of passing the virus to family members, economic impacts), and impacted work relationships among supervisors, colleagues and patients [58]. Many qualitative findings in our study are in line with the findings of the Malaysian study.

Female gender and higher workload were found to be positively associated with burnout in our study, this has been also reported by Matsue et al., who found that female gender, exposure to higher workload as well as the desire for appreciation were all significantly associated with burnout among healthcare workers during the current pandemic [41]. Although the logistic regression model in our study did not find COVID-19 financial incentives as a predicting factor of burnout, many physicians in our qualitative interviews have reported the need for a reliable acknowledgment to HCPs emotionally and financially, which relates to Matsue et al. finding of desire for appreciation. Additionally, female HCPs as well as those who work at high-risk workplaces during the pandemic crisis were found to have significantly higher possibilities of suffering from occupational burnout as reported by Khasne et al. in their recent study [38]. In our study, female physicians, as well as physicians who work at JMOH and JRMS hospitals (highly loaded institutions, labeling them to be risky workplaces), had significantly higher odds of suffering from burnout, and this is consistent with Khasne et al’s findings.

Besides, our study findings regarding predictors of burnout were found to be consistent with many studies that addressed HCPs were exposed to higher levels of psychological distress due to the risk of catching COVID-19 infection and higher workload during the pandemic crisis, making them vulnerable to burnout [39, 40, 59, 60]. Higher workload and long duty hours lead to more exposure to patients, especially among physicians and other HCPs who work directly in managing COVID-19 cases and those who work at emergency units. As of March 30, 2021, a total of 42 physicians have died of COVID-19 in Jordan [61]. Moreover, fear of infection, shortage of healthcare personnel, and adequacy of PPE were reported by physicians in our qualitative interviews to be major factors that afflicted their physical and psychological wellbeing as well as impacting their job satisfaction. This is also consistent with the findings of a recent qualitative study that was conducted in Jordan among a sample of physicians, nurses, and pharmacists during the COVID-19 pandemic [37]. Moreover, other causes of burnout among HCPs amid the COVID-19 pandemic were reported in the literature including unprotected exposure to patients or suspected cases [62], and the lack of physical activity, meditation, or other activities that can increase the resilience of an individual [16]. Those factors were not assessed in our current study as our survey questionnaire and the individual interviews did not assess resilience and coping.

Considering the interconnected concepts between burnout and job satisfaction, many factors at the institutional level and workplaces play important roles in shaping the HCPs’ work engagement, physical and mental wellbeing as well as satisfaction regarding their jobs [18]. In most contexts, working amid the current pandemic crisis was an extraordinary mission for HCPs that required more effort, longer duty hours, and risky sacrifices that exposed them to many job-related stressors [60]. Except for age, job stress/burnout, professional classification, low salary, and highly loaded workplaces (JMOH and JRMS hospitals), were all found to be negatively associated with job satisfaction in our study. These predictors were also addressed in many studies to similarly impact job satisfaction among HCPs during the COVID-19 pandemic [18, 60, 63].

Our present study highlighted a high level of burnout among physicians during the COVID-19 pandemic. In addition, many burnout factors have also afflicted the physicians’ levels of job satisfaction. This could be due to a vicious circle between job-related stress, occupational burnout, and job satisfaction [63]. The COVID-19 pandemic has already impacted most of human life’s domains including, health, politics, economy, education as well as social life [5, 7, 64]. Many pandemic-related measures were believed to negatively impact working conditions, job security, financial security as well as work engagement, leading to severe effects on personal and professional aspects among HCPs and impacting productivity [18, 60]. Additionally, our study findings are expected to be applicable and beneficial in various healthcare settings globally. Although physicians’ and HCPs’ physical and emotional distress, as well as job satisfaction, may differ according to various circumstances in different contexts and settings, the agreement between many findings in our study with various international studies indicates that the impacts of the COVID-19 crisis on HCPs were closely similar despite different cross-border work factors, settings, and preparedness. Consequently, this calls for globally unified efforts to tackle burnout and prevent it along with enhancing work environments by sufficiently addressing the common contributing factors in various settings and advocating appropriate solutions.

Our study findings are expected to provide Jordanian decision-makers with a comprehensive overview regarding contributing factors to burnout and job satisfaction in the context of healthcare services delivery. These factors should be properly taken into consideration to enhance the working conditions of physicians as well as other HCPs as they are a vital cornerstone of the healthcare system. Improving salary scales, reducing duty hours especially for those whose working hours exceed the maximum limit as per Jordanian labor law (48 h/week), creating better opportunities for early-career physicians, expanding the health workforce, and developing pandemic-related unified and clear protocols for HCPs are all expected to help reducing burnout and enhance job satisfaction of physicians and HCPs in general. The battle against the current pandemic is physically and mentally exhaustive to HCPs; thus, decision-makers at the institutional level should also provide emotional, psychological, and financial support to HCPs as an appreciation message. Additionally, the findings of our study reflect the importance of professional and psychological support programs for HCPs in the times of COVID-19 pandemic. Workplace health programs that aim to protect and support the mental wellbeing of healthcare workers are crucial in crises [65, 66]. Workshops that aim at raising awareness about burnout and its prevention methods can be provided by experts targeting physicians and other HCPs along with policymakers and decision-makers of the health sector. Also, training on coping strategies, resilience, and dealing with stress may play important roles in equipping individuals with the required skills to overcome burnout [67]. These workshops can be carried out either face-to-face with respecting the pandemic control measures (i.e., physical distancing) or through synchronous/asynchronous web-based webinars. In addition, organizational support provided by the managerial panel of workplaces is expected to have a positive influence to reduce burnout and enhance job satisfaction [68]. Decision-makers and managers of the health sector in Jordan are advised to consider the previously described crucial factors in future policies and decisions regarding the COVID-19 public health response in the country.

To the best of our knowledge, this is the first mixed-method study that investigated occupational burnout and job satisfaction among physicians in Jordan and the EMR during the COVID-19 pandemic. Our study has many strengths which are shaped in the following facts (i) Implementing a mixed-method study design which helped to gain a comprehensive overview of the topic numerically and narratively, (ii) the power of triangulation between quantitative and qualitative methods, provided by mixed-method research, (iii) having a relatively reasonable sample size in the cross-sectional survey, (iv) using internationally validated scales in the quantitative strand and previously published theoretical models to create the conceptual framework for our qualitative strand, (v) using various techniques as suggested by Lincoln and Guba (1985) [69, 70] to ensure the trustworthiness of the qualitative strand, and lastly (vi) following the Consolidated Criteria for Reporting Qualitative Research (COREQ) and Strengthening the Reporting of Observational Studies in Epidemiology (STROBE) in our qualitative and quantitative strands, respectively (See Additional files 3 and 4).

Nevertheless, some limitations need to be acknowledged and considered as well, including (i) using a convenience sampling strategy in recruiting participants in the cross-sectional survey which might have affected the representativeness of our sample (in terms of sociodemographic and work-related variables) and generalizability of results. However, this was believed to be the only feasible sampling approach considering the current pandemic crisis, (ii) difficulty to assess causal relationships and temporality of events in a cross-sectional design, that is, physicians could have suffered from a significant level of burnout before the pandemic, but these levels were escalated due to pandemic crisis, (iii) these data represent self-reported states, thus, recall bias should be considered (iv) inability to control for multiple survey entries from a technological aspect. However, in our survey’s introductory letter, we asked participants to submit only one response and we did not provide any form of incentives to participants; thus, motivation for filling out the questionnaire more than once was unlikely, (v) our study assessed occupational burnout and job satisfaction among physicians as a subpopulation from HCPs, we encourage for more inclusive studies that involve HCPs such as nurses, technicians, and paramedics, (vi) our study did not assess the specific roles of physicians that were escalated during the pandemic crisis in comparison to pre-pandemic state, and lastly (vii) in the qualitative strand, participants’ body language could not be observed due to using telephone interviews.

## Conclusion

The COVID-19 pandemic has imposed a significant physical and psychological burden on physicians and other HCPs. Although the degree of resilience is inherently different between individuals, many entities under the jurisdiction of healthcare institutions and decision-makers are considered major contributing factors to occupational burnout and play essential roles in job satisfaction, especially in extraordinary situations like the current pandemic crisis. More organized and effective efforts should be implemented aiming at protecting HCPs’ physical and mental wellbeing, enhancing their working conditions, and raising awareness about burnout and how to manage it. The COVID-19 battle has been already exhaustive in terms of efforts and resources, therefore, evidence-based decisions and proper utilization of financial and human resources at the national level are believed to be crucial for the sustainability of the health workforce and healthcare systems especially in contexts with limited resources.

## Supplementary Information

**Additional file 1.** Cross-Sectional Questionnaire.

**Additional file 2.** Interview Guide.

**Additional file 3.** COREQ Statement.

**Additional file 4.** STROBE Statement.

## Data Availability

The raw dataset generated and analyzed in the cross-sectional survey is publicly available on figshare 10.6084/m9.figshare.14058185.v1 [71]. The dataset generated and analyzed in the qualitative interviews is not publicly available due to the potential for individual and organizational privacy to be compromised. Reasonable requests for parts of the qualitative data will be considered by the corresponding author.

## Abbreviations

BMS: Burnout Measure Short Version
HCPs: Health Care Professionals
JMA: Jordanian Medical Association
JDs: Jordanian Dinars
JMOH: Jordanian Ministry of Health
JRMS: Jordanian Royal Medical Services
NGOs: Non-Governmental Organizations
PHs: Private Hospitals
PPE: Personal Protective Equipment
QUAL: Qualitative strand
QUAN: Quantitative strand
SIJS: Short Index of Job Satisfaction
UHs: University Hospitals
WHO: World Health Organization
