# Supplementary material for: Occupational burnout and job satisfaction among physicians in times of COVID-19 crisis: a convergent parallel mixed-method study
Source: BMC Public Health. 2021 Apr 28;21:811. doi: 10.1186/s12889-021-10897-4 (PMC8079229; doi:10.1186/s12889-021-10897-4)
Supplement: Supplementary file 1 — Additional file 1. Cross-Sectional Questionnaire. [file 12889_2021_10897_MOESM1_ESM.pdf]

Occupational Burnout and Job Satisfaction Among Physicians in Times of COVID-19 Crisis: A Convergent Parallel Mixed-Method Study

Dear respected medical doctors in Jordan ,

We hope that you are all safe and in a perfect health.

We would like to provide our extreme appreciation to you and to all healthcare professionals for your immense efforts and sacrifices in fighting the COVID-19 pandemic and working during this extraordinary situation.

We are inviting you to participate in this web-based cross-sectional survey which investigates occupational burnout and job satisfaction among physicians in times of the COVID-19 pandemic in Jordan. This survey is part of a broader mixed-method research study. The survey questionnaire consists of 3 sections with a total of 32 questions (apart from this introductory section). Based on pilot testing , questionnaire requires an average of 7 minutes to be completed. To be eligible to participate in this survey study , you have to be fulfil the following inclusion criteria (both) :

1- Being a medical doctor of any specialty (Medical or Surgical) and of any professional rank (General Practitioner , Resident, Specialist or Consultant) .

2- Practicing your career in Jordan during the COVID-19 pandemic ,whether as frontline or not

Your participation is completely voluntary and you can withdraw at any point of participation without providing reasons . The questionnaire does not require any personal information; thus, your identity is anonymous and the collected data will be stored securely and only used for research purposes . You are kindly requested to read the questions carefully , to answer all items, and to submit it only once. You can share the link of this survey with your workmates who are medical doctors in Jordan as well.

Our research project was approved by ethics committee at Faculty of Medicine , Mutah University (Ref# 1112020).We appreciate your valuable and precious contribution and participation that will let us as health researchers to get a comprehensive overview of this topic , considering that findings are expected to provide managers and decision makers of the health sector in Jordan with important scientific insights into the current situation of medical practice amid COVID-19 pandemic .

For any further enquiry , do not hesitate to contact the principal investigators of this project :

1-Hamzeh Al-Rawashdeh , MD, JBOpht
Email : dr_hmsr@yahoo.com
Mobile : +962 7 9727 4355

2-Ala'a B. Al-Tammemi , MD, MScPH
Email: alaa.tammemi@med.unideb.hu
Mobile : +36 20 332 1362

*Required

1. Eligibility Question : I confirm that I am a Medical Doctor working in Jordan during the COVID-19 pandemic crisis. Also, I consent to participate in this survey voluntarily *

   Mark only one oval.

   - Yes
   - No

Sociodemographic & Work Profile

Please answer these items which describe your sociodemographic and work profile

2. 1-Please write down your age using Arabic numerals *

   ____

3. 2-Please choose your gender *

   Mark only one oval.

   - Male
   - Female
   - Prefer not to say

4. 3-Please choose your current marital status *

Mark only one oval.

- Single (Never married, separated , Widowed , Divorced)
- Married

5. 4-Please choose the number of persons you currently live with in the same house/residence. *

Mark only one oval.

- 1-3 persons
- 4-6 persons
- >6 parsons

6. 5- Please choose the type of your residence/living place *

Mark only one oval.

- Rented Flat/house
- Owned Flat/house

7. 6- Where do you live in Jordan? *

Mark only one oval.

- Northern region (Irbid, Jerash, Ajloun , Mafraq)
- Central region (Amman, Zarqa, Madaba, Balqa'a)
- Southern region (Karak, Tafileh, Ma'an , Aqapa)

8. 7-Please , choose your current smoking status *

Mark only one oval.

- ( ) Smoker
- ( ) Non-smoker

9. 8-What is your specialty discipline? You can add your specialty discipline if not found among choices *

Mark only one oval.

- General Practice / Primary Health Care/ Internship
- General Internal Medicine
- General Surgery
- Obstetrics & Gynecology
- Family Medicine
- General Pediatrics
- Public Health/Community Medicine/Preventive Medicine
- Hematology/Oncology
- Neurosurgery
- Ophthalmology
- Neurology
- Nephrology
- Infectious Diseases
- Dermatology
- Anesthesia and ICU
- Emergency Medicine
- Cardiology
- Cardiothoracic Surgery
- Otorhinolaryngology (ENT)
- Urology
- Vascular Surgery
- Gastrointestinal Surgery
- Pediatric Surgery
- Radiology / Nuclear Medicine / Radiation Oncology
- Endocrinology
- Gastroenterology
- Laboratory Medicine / Pathology / Microbiology
- Psychiatry
- Rheumatology

Aviation/Aerospace Medicine

Orthopedics

Other: ______________________________

10. 9-What best describes your current professional classification according to Jordanian laws? *

Mark only one oval.

- General Practitioner (GP) , including Internship doctors
- Resident Doctor
- Specialist Doctor
- Consultant Doctor

11. 10-Where do you currently work? *

Mark only one oval.

- Ministry of Health Hospitals
- Non-Governmental Organizations (NGOs)
- Royal Medical Services (RMS) Hospitals
- University Hospitals
- Private Hospitals/Clinics

12. 11-What is your current Job status? *

Mark only one oval.

- Full-Time
- Part-Time

13. 12-What is your average monthly salary? *

Mark only one oval.

- ( ) <700 JDs
- ( ) 700-1400 JDs
- ( ) >1400 JDs

14. 13-What is your average duty hours/week during the COVID-19 pandemic crisis? *

Mark only one oval.

- ( ) <40 hours/Week
- ( ) 40-48 hours/Week
- ( ) >48 hours/Week

15. 14-How many night duties/week do you preform? *

Mark only one oval.

- ( ) No night duty
- ( ) 1-3
- ( ) >3

16. 15-Have you been provided with adequate supply of Personal Protective Equipment for your own use at your work ? (Masks , Hand sanitizers , Gowns , Goggles...etc) *

Mark only one oval.

- ( ) Yes
- ( ) No

17. 16-Have you received an Overtime/Incentives payment during your work in the COVID-19 crisis? *

Mark only one oval.

- Yes
- No

18. 17-Have you been tested positive for SARS-CoV-2 ? *

Mark only one oval.

- Yes
- No

Burnout

Please , answer the following items which assess different aspects of physical , emotional and psychological wellbeing at work . Please note that the answers are rated , choose the best choice that applies to your situation .

19. 18- When you think about you [redacted]

Mark only one oval.

- Never
- Almost Never
- Rarely
- Sometimes
- Often
- Very Often
- Always

20. 19- When you t [redacted]
DISAPPOINTED [redacted]

Mark only one oval.

- Never
- Almost Never
- Rarely
- Sometimes
- Often
- Very Often
- Always

21. 20- When you think about your [redacted]

Mark only one oval.

- Never
- Almost Never
- Rarely
- Sometimes
- Often
- Very Often
- Always

22. 21- When [redacted]
TRAPPED/ [redacted]

Mark only one oval.

- Never
- Almost Never
- Rarely
- Sometimes
- Often
- Very Often
- Always

23. 22- When you think about your [redacted]

Mark only one oval.

- Never
- Almost Never
- Rarely
- Sometimes
- Often
- Very Often
- Always

24. 23- When you think about your [redacted]

Mark only one oval.

- Never
- Almost Never
- Rarely
- Sometimes
- Often
- Very Often
- Always

25. 24- When you think about your [redacted]
WEAK? *

Mark only one oval.

- Never
- Almost Never
- Rarely
- Sometimes
- Often
- Very Often
- Always

26. 25- Wh [redacted]
LIKE A [redacted]

Mark only one oval.

- Never
- Almost Never
- Rarely
- Sometimes
- Often
- Very Often
- Always

27. 26- When y [redacted]
WITH SLEEP [redacted]

Mark only one oval.

- Never
- Almost Never
- Rarely
- Sometimes
- Often
- Very Often
- Always

28. 27- When [redacted]
TOLERATE

Mark only one oval.

- Never
- Almost Never
- Rarely
- Sometimes
- Often
- Very Often
- Always

Job Satisfaction

Please , answer the following items which assess different aspects of your perception and attitudes toward your current job . Please note that the answers are rated , choose the best choice that applies to your situation .

29. 28- I feel fai [redacted]

Mark only one oval.

- Strongly disagree
- Disagree
- Neutral
- Agree
- Strongly agree

30. 29- Most days I a [redacted]

Mark only one oval.

- Strongly disagree
- Disagree
- Neutral
- Agree
- Strongly Agree

31. 30- Each day at work [redacted]

Mark only one oval.

- Strongly disagree
- Disagree
- Neutral
- Agree
- Strongly agree

32. 31- I find real [redacted]

Mark only one oval.

- Strongly disagree
- Disagree
- Neutral
- Agree
- Strongly agree

33. 32- I consider m [redacted]

Mark only one oval.

- Strongly disagree
- Disagree
- Neutral
- Agree
- Strongly Agree

This content is neither created nor endorsed by Google.

Google Forms
