# Supplementary material for: Occupational burnout and job satisfaction among physicians in times of COVID-19 crisis: a convergent parallel mixed-method study
Source: BMC Public Health. 2021 Apr 28;21:811. doi: 10.1186/s12889-021-10897-4 (PMC8079229; doi:10.1186/s12889-021-10897-4)
Supplement: Supplementary file 2 — Additional file 2. Interview Guide. [file 12889_2021_10897_MOESM2_ESM.pdf]

SEMI-STRUCTURED INTERVIEW GUIDE

1- Please describe your feelings after a day at work during the COVID-19 pandemic.

2- Please describe how satisfied you are with working conditions during the pandemic crisis.

3- In your opinion, mention and describe what factors have negatively impacted your work and your work-related satisfaction during the current pandemic.

4- In your opinion, mention and describe what factors have motivated you to work during the current pandemic crisis.

5- Please describe any procedures or protocols that have been implemented by decision-makers at your institution and/or at the national level to enhance your working conditions during the current pandemic crisis.

6- Please describe how did lockdown and curfew policy in Jordan impact your medical practice, as well as your personal life? Please provide us with detailed experiences.
