# Supplementary material for: Occupational burnout and job satisfaction among physicians in times of COVID-19 crisis: a convergent parallel mixed-method study
Source: BMC Public Health. 2021 Apr 28;21:811. doi: 10.1186/s12889-021-10897-4 (PMC8079229; doi:10.1186/s12889-021-10897-4)
Supplement: Supplementary file 3 — Additional file 3. COREQ Statement. [file 12889_2021_10897_MOESM3_ESM.pdf]

Consolidated criteria for reporting qualitative studies (COREQ): 32-item checklist

Developed from:
Tong A, Sainsbury P, Craig J. Consolidated criteria for reporting qualitative research (COREQ): a 32-item checklist for interviews and focus groups. *International Journal for Quality in Health Care*. 2007. Volume 19, Number 6: pp. 349 – 357

No. Item	Guide questions/description	Reported on Page #
**Domain 1: Research team and reflexivity**		
Personal Characteristics		
1. Inter viewer/facilitator	Which author/s conducted the inter view or focus group?	Page 13,
2. Credentials	What were the researcher's credentials? E.g. PhD, MD	Page 13
3. Occupation	What was their occupation at the time of the study?	Page 13
4. Gender	Was the researcher male or female?	Page 13
5. Experience and training	What experience or training did the researcher have?	Page 13
Relationship with participants		
6. Relationship established	Was a relationship established prior to study commencement?	N/A
7. Participant knowledge of the interviewer	What did the participants know about the researcher? e.g. personal goals, reasons for doing the research	N/A
8. Interviewer characteristics	What characteristics were reported about the inter viewer/facilitator? e.g. Bias, assumptions, reasons and interests in the research topic	N/A
**Domain 2: study design**		
Theoretical framework		
9. Methodological orientation and Theory	What methodological orientation was stated to underpin the study? e.g. grounded theory, discourse analysis, ethnography, phenomenology, content analysis	Methods, Page 12-13
Participant selection		
10. Sampling	How were participants selected? e.g. purposive, convenience, consecutive, snowball	Methods, Page 12
11. Method of approach	How were participants approached? e.g. face-to-face, telephone, mail, email	Methods, Page 12
12. Sample size	How many participants were in the study?	Results, page 18
13. Non-participation	How many people refused to participate or dropped out? Reasons?	N/A

Setting		
14. Setting of data collection	Where was the data collected? e.g. home, clinic, workplace	Methods, Page 12
15. Presence of non-participants	Was anyone else present besides the participants and researchers?	N/A
16. Description of sample	What are the important characteristics of the sample? e.g. demographic data, date	Results, Page 18
Data collection		
17. Interview guide	Were questions, prompts, guides provided by the authors? Was it pilot tested?	Methods, Page 12
18. Repeat interviews	Were repeat inter views carried out? If yes, how many?	N/A
19. Audio/visual recording	Did the research use audio or visual recording to collect the data?	Methods, Page 13
20. Field notes	Were field notes made during and/or after the inter view or focus group?	N/A
21. Duration	What was the duration of the inter views or focus group?	Methods, Page 13
22. Data saturation	Was data saturation discussed?	Methods, Page 13
23. Transcripts returned	Were transcripts returned to participants for comment and/or correction?	N/A
**Domain 3: analysis and findings**		
Data analysis		
24. Number of data coders	How many data coders coded the data?	Methods, Page 14
25. Description of the coding tree	Did authors provide a description of the coding tree?	N/A
26. Derivation of themes	Were themes identified in advance or derived from the data?	Methods, Page 14
27. Software	What software, if applicable, was used to manage the data?	NVivo, Page14
28. Participant checking	Did participants provide feedback on the findings?	N/A
Reporting		
29. Quotations presented	Were participant quotations presented to illustrate the themes/findings? Was each quotation identified? e.g. participant number	Results, Pages 18-28
30. Data and findings consistent	Was there consistency between the data presented and the findings?	Relationship to existing knowledge
31. Clarity of major themes	Were major themes clearly presented in the findings?	Results, Page 18
32. Clarity of minor themes	Is there a description of diverse cases or discussion of minor themes?	N/A
